# Innovative Approach to Accelerate Wound Healing: Synthesis and Validation of Enzymatically Cross-Linked COL–rGO Biocomposite Hydrogels

**DOI:** 10.3390/gels10070448

**Published:** 2024-07-06

**Authors:** Luisbel González, Víctor Espinoza, Mauricio Tapia, Valentina Aedo, Isleidy Ruiz, Manuel Meléndrez, Claudio Aguayo, Leonard I. Atanase, Katherina Fernández

**Affiliations:** 1 Laboratorio de Biomateriales, Departamento de Ingeniería Química, Facultad de Ingeniería, Universidad de Concepción, Concepción 4030000, Chile; luisbgonzalez@udec.cl (L.G.); vespinozar@udec.cl (V.E.); mtapia2017@udec.cl (M.T.); vaedo2017@udec.cl (V.A.); isruiz@udec.cl (I.R.); 2 Facultad de Ciencias para el Cuidado de la Salud, Universidad San Sebastián, Campus Las Tres Pascualas, 20Lientur 1457, Concepción 4060000, Chile; manuel.melendrez@uss.cl; 3 Departamento de Bioquímica Clínica e Inmunología, Facultad de Farmacia, Universidad de Concepción, Concepción 4030000, Chile; caguayo@udec.cl; 4 Faculty of Medicine, “Apollonia” University of Iasi, 700511 Iasi, Romania; 5 Academy of Romanian Scientists, 050045 Bucharest, Romania

**Keywords:** wound healing, collagen, rGO, dopamine, enzymatic cross-linking

## Abstract

In this study, an innovative conductive hybrid biomaterial was synthetized using collagen (COL) and reduced graphene oxide (rGO) in order for it to be used as a wound dressing. The hydrogels were plasticized with glycerol and enzymatically cross-linked with horseradish peroxidase (HRP). A successful interaction among the components was demonstrated by FTIR, XRD, and XPS. It was demonstrated that increasing the rGO concentration led to higher conductivity and negative charge density values. Moreover, rGO also improved the stability of hydrogels, which was expressed by a reduction in the biodegradation rate. Furthermore, the hydrogel’s stability against the enzymatic action of collagenase type I was also strengthened by both the enzymatic cross-linking and the polymerization of dopamine. However, their absorption capacity, reaching values of 215 g/g, indicates the high potential of the hydrogels to absorb fluids. The rise of these properties positively influenced the wound closure process, achieving an 84.5% in vitro closure rate after 48 h. These findings clearly demonstrate that these original composite biomaterials can be a viable choice for wound healing purposes.

## 1. Introduction

Wound healing is a biological process of high complexity oriented to accomplishing the recovery of a tissular lesion. Generally, wound dressings such as gauze, bandages, or cotton are used to keep the wound isolated, but they do not induce skin regeneration [1,2]. In this context, an interesting option is to use dressings based on biomaterials, which should be non-toxic and biocompatible, allowing gas interchange while protecting the wound from mechanical stress [3,4,5,6,7,8].

Hydrogels are generally biocompatible and biodegradable materials having a tridimensional network and a high swelling capacity owing to their cross-linked structure. Collagen (COL)-based hydrogels have the potential to be used as wound dressings [9,10]. However, their mechanical properties are not suitable for their use as patches and, therefore, biocomposite hydrogels need to be prepared.

Graphene oxide (GO) is a nanomaterial with excellent mechanical, thermal, and rheological properties [11,12,13]. Nevertheless, the high number of oxygen-containing hydrophilic groups makes GO unstable [14]. These oxygen-functional groups, considered toxic, have been commonly reduced using hydrazine, dimethylhydrazine, hydroquinone, and sodium borohydride compounds [15,16]. Functional group passivation provides reduced graphene oxide (rGO), a compound with superior conductivity to GO; nevertheless, in the absence of a suitable micro or macromolecular dispersant, rGOs have a tendency to agglomerate irreversibly through van der Waals interaction [17]. The use of soft or green agent reductors such as Dopamine (DA) can increase rGO stability and dispersion [16,17]. At alkaline pH, DA auto-polymerizes and forms a polydopamine (PDA) coating on the rGO’s surface [18]. Moreover, PDA-rGO has been demonstrated to be a platform that can be functionalized with natural polymers [19].

rGO incorporation into a COL matrix has been used to produce scaffolds for tissue engineering [20], for wound healing of injured diabetics [21,22], osteogenic differentiation of human mesenchymal stem cells [23], and as an osteoinductive extracellular matrix for the repair of cranial defects in rats [24]. To the best of our knowledge, the composite COL–rGO hydrogels have not been used as a wound dressing.

Although COL–rGO hydrogels have been developed for different applications, no studies exist concerning the simultaneous use of DA as a GO green reductor agent or horseradish peroxidase (HPR) as cross-linking agent, in order to improve the material’s physicochemical and biological properties. The enzymatic cross-linking, in the presence of HRP and catalyzed by H_2_O_2_, induces covalent bonding between COL and rGO. Additionally, the DA polymerization generates strong cross-linking bonds in the hydrogel matrix. DA phenolic groups can oxidize and auto-conjugate through C–C bonds between the ortocarbon of the aromatic ring or through C–O bonds between the ortocarbon and the phenolic oxygen. This mechanism has previously been investigated with favorable results regarding hydrogel processability [25].

The aim of this study was to develop a conductive biocomposite hydrogel COL–rGO, using the system HPR/H_2_O_2_, that can enhance wound cicatrization. To this end, the influence of rGO concentration on the physicochemical and biological properties of the hydrogel was assessed by different techniques.

## 2. Results and Discussion

### 2.1. Morphological Characterization of COL–rGO Hydrogels

**Figure 1:**
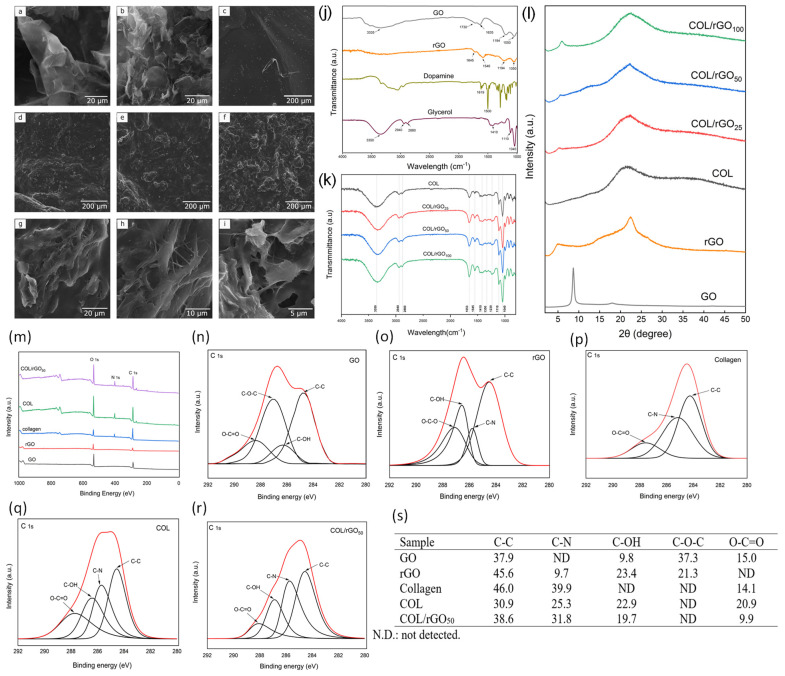
Morphological and chemical characterization of hydrogels. SEM images of (**a**) GO, (**b**) rGO, (**c**) COL hydrogel, (**d**) COL/rGO_25_, (**e**) COL/rGO_50_, (**f**) COL/rGO_100_, and (**g**–**i**) COL/rGO_50_ at different scales. FTIR Spectra of (**j**) GO, rGO, DA, and glycerol; (**k**) COL hydrogel, COL/rGO_25_, COL/rGO_50_ y COL/rGO_100_ hydrogels. (**l**) X-ray diffraction patterns of GO, rGO, and the synthesized COL hydrogel, COL/rGO_25_, COL/rGO_50_ y COL/rGO_100_ samples. Surface composition of the samples (**m**) XPS survey spectrum of GO, rGO, collagen, COL hydrogel, and COL/rGO_50_. Deconvoluted C 1S XPS spectra of (**n**) GO, (**o**) rGO, (**p**) pure collagen, (**q**) COL hydrogel, (**r**) COL/rGO_50_. (**s**) Percentage in area of maximum components with binding energies of all samples.

The samples’ morphology was analyzed by scanning electron microscopy (SEM), and the micrographs are shown in Figure 1a–i. The GO SEM image (Figure 1a) reveals a structure of wrinkled and randomly aggregated layers, with the absence of flat surfaces and with orderly layer structures characteristic of graphite, which is a product of the oxidation. The SEM image of rGO (Figure 1b) reveals a decrease in size and an increase in the number of wrinkled sheets, with a more disordered structure than GO and a tendency to become entangled with each other, caused by van der Waal interactions [26].

The hydrogels’ morphology is observed in Figure 1c–f, where an increasing rGO amount produced a significant growth in surface irregularity, which could induce a larger contact area between cells and the material. In Figure 1g,i, the presence of collagen fibers was noticed, which increases the porosity of the hydrogel and thus the fluid adsorption. 

### 2.2. Chemical Characterization of COL–rGO Hydrogels

FTIR spectroscopy was used to identify the chemical structure of GO, rGO, DA, glycerol, COL, and COL–rGO hydrogels. The GO spectrum (Figure 1j) shows the bands associated with the oxygenated groups, the vibration of the surface hydroxyl groups (C−OH) being visible, in addition to the absorbed water molecules in a broad band at 3335 cm^−1^.The bands related to C=O (−COOH) vibrations are visible at 1730 cm^−1^, and the stretching modes of the epoxy (C−O−C) and alkoxy (C−O) groups are observed at 1194 and 1050 cm^−1^, respectively. The band at 1635 cm^−1^ can be attributed to the aromatic double bond (C=C) vibrations of the sp^2^ carbon skeletal lattice and to the C−H bending vibration frequencies of the carbon lattice [16,27].

The DA spectrum has bands at 1619 and 1500 cm^−1^, corresponding to the C=C stretching vibration of the benzene ring and the −OH stretching vibration of the DA diols [28]. In the rGO spectrum (Figure 1j), the bands associated with the stretching vibrations of the C=O (amide I) groups appear at 1645 cm^−1^ and the N−H bending and C−H stretching vibrations (amide II) at 1546 cm^−1^. A decrease in the intensity of the bands associated with the epoxy and alkoxy groups is observed at 1194 and 1050 cm^−1^, respectively. These elements demonstrate the successful reduction in GO with DA and, simultaneously, the rGO functionalization by DA amino groups.

The FTIR spectrum of COL–rGO (Figure 1k) shows the characteristic bands corresponding to amides I, II, and III at 1653, 1545, and 1239 cm^−1^, respectively [29]. A light change of amide I to a higher frequency (1645 to 1653 cm^−1^) can be observed, since the C=O stretching vibrations of rGO and amide I of COL occur in this band. The peak at 1410 cm^−1^ is attributed to the C−OH bonds of the catechol groups from DA, indicating that DA has been successfully introduced into the COL hydrogel [30]. A peak around 1350 cm^−1^ is observed in all hydrogels, which can be attributed to a stretching of the indole ring, supporting the formation of 5,6-dihydroxyindole/5,6-indolequinone units that are characteristic of COL-DA hydrogels [30].

The broad band between 3200 and 3600 cm^−1^ is caused by the –OH groups stretching of the collagen [31]. Moreover, the peak at 3350 cm^−1^ can be specifically attributed to the presence of hydrogen bonds formed between intermolecular water, glycerol, and collagen amide A. These hydrogen bonds are associated with the stretching vibrations of the N–H groups [30,32].

It was determined that varying concentrations of rGO have no significant impact on the absorbance of amide A. This finding suggests that the water molecules bound to the collagen were not lost, enabling the formation of new bonds with different molecules. The characteristic bands of glycerol can be observed at 1040 and 1110 cm^−1^, indicating the stretching of C−O bonds in primary and secondary alcohols, respectively [33]. The spectral region between 2800 and 3000 cm^−1^ corresponds to the bands linked to C−H aliphatic bonds. These bands can be attributed to the presence of glycerol lipids found in the hydrogel [32].

An increase in the intensity associated with the amide bands (I, II, and III) and a disappearance of the peak at 1450 cm^−1^ were observed when increasing the amount of rGO to the hydrogel, which can be assigned to the bending vibrations of CH_2_ [34,35]. These differences in intensity suggest the formation of new hydrogen bonds among the hydroxyl, amino, and carboxylic groups [36].

The effect of the components grafted onto the hydrogel on the stability of collagen can be evaluated through the amide I profile, where the α triple helix band is observed at 1650 cm^−1^ [36], and the formation of the β sheet was not perceived [37]. COL hydrogel’s stability was calculated through the relation between the amide III and the aliphatic band of bonds C–H (1456 cm^−1^) [38]. This relation was higher than 1 for all synthetized materials, showing that rGO grafting did not affect the α triple helix structure.

The cross-linking degree is a key aspect that must be controlled in hydrogels since it is directly related to their pore size and absorption capacity [39]. The ratio between amide A/amide I was used as a collagen reticulation measure, revealing that increased rGO favors the cross-linking density.

The hydrogels’ crystallinity was evaluated through XRD patterns (Figure 1l). The XRD pattern of the GO revealed a very sharp diffraction peak at 2θ = 8.68° and an interlayer distance of 9.86 Å; these elements demonstrate the complete oxidation of graphite and the intercalation of oxide functional groups on the carbon basal plane, such as epoxy, hydroxyl, carbonyl, and carboxyl groups [40,41]. After the GO reaction with DA, a wide pick was obtained (18° to 28°) with a maximum intensity at 2θ = 22.5, reducing the interplanar distance to 3.96 Å. These findings can be attributed to the elimination of oxygenated functional groups and the π−π stacking among the hexagonal cells of graphene and the DA aromatic ring, showing a favorable GO reduction [16,17].

The COL hydrogel presented three characteristics peaks within its structure: the first one at 6° shows the intermolecular side packing among the collagen molecular chains; then, at 20° there is the diffuse association of collagen; and the third peak, at 33°, is associated with the residual amino acid of the triple helix [42,43,44]. Moreover, the diffractograms of the samples revealed a scattered distribution of collagen fibers, with a distinct peak of rGO at 6°. The intensity of the peak increases as the concentration of rGO rises. The peak centered around 2θ = 22° shows a proportional increment in intensity corresponding to the amount of rGO. This can be attributed to the masking effect on the collagen spectrum and the emergence of the distinctive rGO peak. The disappearance of the peak at 33° may be attributed to interference caused by the presence of a highly crystalline structure, like rGO.

The surface compositions of GO, rGO, pure collagen, COL hydrogel, and COL/rGO_50_ were studied by XPS (Figure 1m). It appears that carbon (C1s) and oxygen (O1s) were the predominant components in all the systems, with binding energies of 284.6 and 531.9 eV, respectively. Furthermore, nitrogen (N1s) was detected in the rGO, pure collagen, COL, and COL/rGO_50_, with a binding energy of 399.7 eV.

The impact of DA reduction was assessed by comparing the oxygenated group content in GO and rGO through the calculation of the areas under the C1s and O1s peaks. The C/O ratio of GO was found to be lower (2.37/1) as compared to rGO (4.96/1), indicating the successful reduction in oxygenated functional groups in GO. Similar values for the C/O ratio have been obtained in other studies, with GO and rGO exhibiting ratios of 2.75/1 and 5.2/1, respectively [19]. The C1s deconvolution spectra of GO and rGO (Figure 1n,o) enabled the identification of the oxygenated groups that were most extensively eliminated. The positions of the GO peaks were 284.6 eV (C=C, sp^2^-hybridized carbon, and C–C, sp^3^-hybridized carbon), 286.5 eV (C–OH), 286.9 eV (C–O–C), and 288.2 eV (O–C=O) [19]. In the deconvolution of the rGO peaks, changes can be noticed. The C–C bond maintains its position at 284.6 eV, while the C–N bond is observed at 285.9 eV. The presence of the C–N bond can be attributed to the combination of the DA amino group and the carbon atoms in rGO. These findings were consistent with the observations made in the FTIR analysis. Additionally, the C–OH and C–O–C bonds are now centered at 266.7 and 287.1 eV, respectively. The observed increase in binding energies can be attributed to the removal of the oxygenated functional groups present in GO, which had a shielding effect on the valence electrons. This indicates a higher concentration of carbon atoms with more robust bonds, as depicted in Figure 1s. Following the reduction process, there was an increase in the percentage of C–OH bonds from 9.8% to 23.4% on rGO. This increase can be attributed to the presence of hydroxyl groups associated with the aromatic ring of DA.

The deconvolution of C1s in pure collagen (Figure 1p) revealed distinct peaks located at 284.6, 285.9, and 288.2 eV. These peaks can be associated with C–C, C–N, and O–C=O bonds, respectively [23]. The combination of DA and glycerol (Figure 1q), along with the catalytic action of HRP/H_2_O_2_ within the hydrogel sample, leads to the formation of C–OH bonds and a rise in O–C=O bonds. The presence of C–OH bonds is primarily due to the DA catechol groups, rather than glycerol. This is because glycerol is typically absorbed into the hydrogel structure and does not remain on the surface [45]. The inclusion of rGO (Figure 1r) favors the formation of C–N bonds, supporting the FTIR findings that suggest the creation of amide bonds with collagen. Furthermore, the potential partial oxidation of rGO by HRP/H_2_O_2_ in the hydrogel matrix is unlikely to be based on the observed decrease in the concentration of C–OH and O–C=O bonds. Therefore, the observed increase in the intensity of the peak with lower binding energy indicates a successful interaction of collagen in the synthesis process [46].

### 2.3. Thermal Stability of COL–rGO Hydrogels

The thermal stability of the hydrogel samples was analyzed by thermogravimetric analysis (TGA) and differential thermogravimetric analysis (DTG) (Figure S1 and Table 1). In all synthesized samples, the thermograms displayed two distinct decomposition processes. The first occurred between 83 and 115 °C, which indicated the evaporation of water. The second decomposition step took place between 184 and 337 °C, being related to the deterioration of the collagen fibers. The increase in rGO concentration raised the degradation temperature associated with moisture loss. The first inflection in the curve refers to the water absorbed, indicating that the polymers are sensitive to moisture absorption, which may increase the swelling capacity of hydrogels.

Specifically, the collagen thermal degradation started at 225 °C, with a main weight loss at 286 °C, a value lower than the decomposition range reported for native collagen (300–400 °C) [47]; this difference could be caused by the loss of low molecular weight proteins and the presence of glycerol in the hydrogel. Other studies working with collagen film and glycerol reported a degradation temperature of 223 °C [38], values even lower to those obtained here. This thermal strengthening could be caused by an increase in the cross-linking density generated by the HRP/H2O2 and the polymerization of DA. Studies have shown that the collagen triple helices are stabilized by numerous covalent bonds generated by HRP, which increases the denaturation temperature [48,49].

The COL hydrogel decomposition temperature was 225 °C, and the rGO incorporation lowered it to 211, 193, and 184 °C for COL/rGO25, COL/rGO50, and COL/rGO100, respectively. These changes indicate that enzymatic and chemical cross-linking between the polymers occurring in the hydrogel matrix. The observed decrease in heat released during degradation can be attributed to the high thermal conductivity of rGO. In fact, the incorporation of rGO in the hydrogels facilitates the dissipation of heat produced during the degradation process. Moreover, the residual carbon content increases with increasing rGO concentration. Thus, the thermograms suggest that the rGO was distributed in the hydrogel and that the obtained biocomposite hydrogels were thermally stable under the working conditions.

### 2.4. Swelling Capacity, Penetration Mechanism, and Wettability

**Figure 2:**
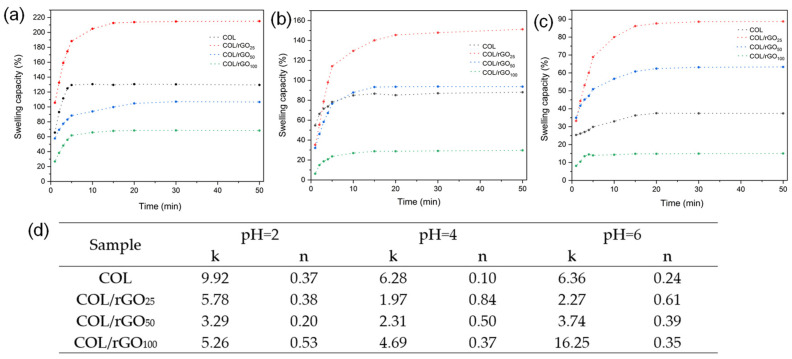
Swelling capacity of COL hydrogel, COL/rGO_25_, COL/rGO_50_ and COL/rGO_100_ at (**a**) pH = 2, (**b**) pH = 4, and (**c**) pH = 6. (**d**) Swelling kinetic parameters for different hydrogels using the Power Law.

The swelling capacity of the materials was determined in PBS at different pH values in order to assess their anionic or cationic behavior. The absorption curves (Figure 2a–c) reached the equilibrium state after 15 min, for all samples, with different maximum values. The hydrogel sample with the highest absorption capacity was COL/rGO_25_, reaching 215, 151, and 88 g/g at pH values of 2, 4, and 6, respectively. The swelling capacity of the materials increases considerably when the medium pH decreases, evidencing the anionic characteristics of the materials, with a predominance of protonatable functional groups. Moreover, the hydrogel’s absorption capacity decreased with the increasing rGO concentration (from 25 to 100 g). The observed phenomenon can be attributed to the preservation of the hydrogel’s original morphology by the rGO and the occupation of the hydrogel’s pores. The kinetic parameters obtained after adjusting the Fick Law to the experimental data are presented in Figure 2d. Most of the hydrogels had an exponent n of less than 0.5, indicating Fickian diffusion. Values over 0.5 indicate the presence of abnormal transport, where an overlap of molecular diffusion and stress relaxation may occur during swelling [50]. A decrease in the values of n can be observed at pH = 4 and pH = 6, with increasing rGO concentration, which is attributed to a protective effect of rGO on collagen fibers and a lower probability of their relaxation. This wide range of values indicates the different swelling mechanisms of the synthesized hydrogels. Additionally, the differences between the k values are due to a different water intake rate in the synthesized materials. These elements could suggest a heterogeneity in the pore size as a function of the rGO concentration.

Contact angles of the rGO, GO, and COL hydrogels and the COL–PDA–rGO hydrogel were used as a measure of the hydrogel’s wettability (Table 2). The contact angles of GO and rGO were 61.8 and 84.5°, respectively. These values were similar to those obtained in other studies [39,51]. This increase may be caused by the elimination of functional groups present in the GO during the reduction process, making the surface more hydrophobic. The contact angle of the COL hydrogel was 49.8°, it being more hydrophilic than both GO and rGO. This difference in the sample hydrophilicity can be attributed to the unique behavior of collagen’s triple helices, which allows the absorption and retention of water [52].

All the hydrogel samples showed a contact angle of less than 90°. The incorporation of DA, glycerol, and HRP/H_2_O_2_ into the collagen increased the contact angle from 49.8 to 50.6° for the COL sample, probably caused by the formation of bonds among the components, increasing the cross-linking collagen density. As expected, increasing the rGO concentration enhances the hydrophobicity of the material (Table 2). This can be attributed to the presence of a limited number of sites that can form hydrogen bonds with water. These transformations in the hydrogel hydrophobicity indicate changes in the chemical surface and are the consequence of a successful chemical/enzymatic reaction.

### 2.5. Conductivity and Surface Charge

The method used to determine the electrical resistance of the materials was the 4-point method. Table 2 shows the conductivity values of the materials determined from the resistance measured. The values fluctuated between 20.02 and 39.76 mS/m, where the conductivity of hydrogels is directly correlated with rGO concentrations, which can be attributed to the creation of an additional electric field [53]. It is important to note that the electrical conductivity of human skin falls within the range of 10^−2^ to 260 mS/m [54]. Scientific studies have shown that using conductive dressings with similar conductivity values to the skin enhances the effective transmission of electrical signals and directs various cellular mediators, such as platelets, inflammatory cells, cytokines, growth factors, and matrix metalloproteinases, towards the center of tissue injuries, thus facilitating tissue repair [55,56,57,58].

The conductivity values of the COL hydrogel were found to be significantly higher than those achieved by Norahan et al. [59] in their study on COL/rGO hydrogels with a ratio of 1:0.08, who reported conductivity values of only 0.12 mS/m. The highest conductivity values obtained may be caused by the presence of nitrogenous groups in the collagen structure, which in the presence of water lose an electron, acquiring the ability to accept electrons, thus facilitating their movement [60]. The glycerol influence was also detected; this compound has a large number of hydroxyl groups that increase water retention and reduce the intermolecular forces between the polymer chains, improving electrical and mechanical properties [61]. Finally, the high humidity of the hydrogels was a parameter that could also favor their conductivity, since the auto-ionization of the water creates a constant ionic flow in the hydrogel matrices.

The surface charge of the materials was determined by DLS. The GO surface charge was −32.8 ± 2.3 mV, and after DA reduction it reached a value of −70.2 ± 5.4 mV (Table 2). The negative values observed for GO can be attributed to the ionization of carboxyl groups, indicating that the oxidation reaction in the synthesis process was appropriately washed [51]. The removal of oxygenated functional groups during reduction restores the electronic configuration of the graphene structure and leads to a higher density of free electrons available for charge transfer [62]. The surface charge of the hydrogels varied from 4.5 to −79.0 mV, suggesting that the presence of glycerol, DA, and rGO alters the surface properties (Table 2). The incorporation of DA and glycerol into the matrix caused a decrease in surface charge from 4.5 ± 0.8 to 9.2 ± 0.4 mV. This change can be attributed to the neutralization of carboxyl and amino groups as a result of their interaction with these compounds. The slight variation observed indicates that these compounds did not affect the helical structure of collagen. The rGO inclusion into the matrix resulted in a noticeable reduction in the surface charge, which could be attributed to the deprotonation of the collagen’s carboxyl groups upon interaction with rGO, as well as the ability of rGO to absorb negatively charged species into the solution [62].

An additional study examined the impact of pH on the surface charge of dissolved collagen (Table S1). When the collagen was dissolved in HCl (0.05 M, pH = 4), the zeta potential measurements showed values of 12.3 ± 0.4 mV. This change in surface charge can be explained by the protonation of carboxyl groups, specifically those in glutamic acid and aspartic acid, within the collagen structure [63]. As the pH increased, a noticeable decrease in surface charge was observed, reaching a value of −13.5 ± 0.2 mV at pH = 9, which can be attributed to the deprotonation of carboxyl groups [63,64].

### 2.6. Biodegradability and Cytotoxicity Assays

**Figure 3:**
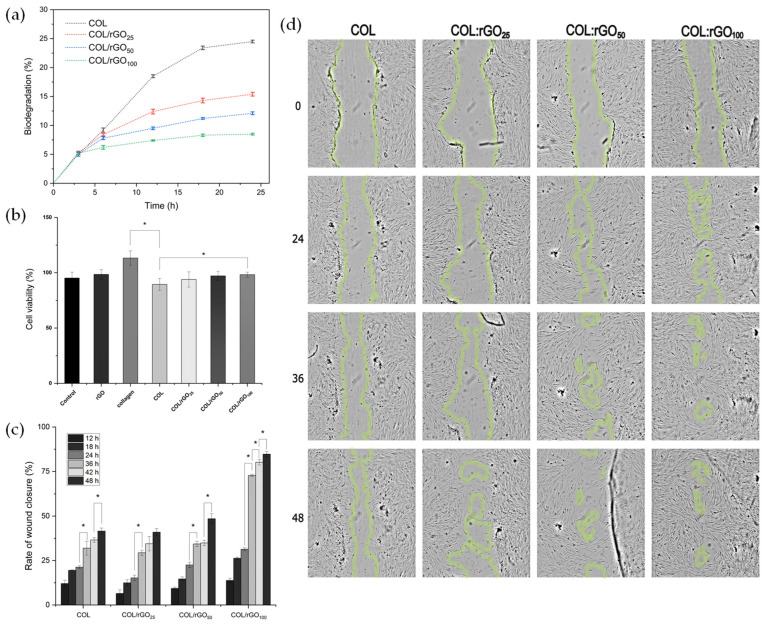
(**a**) Biodegradation performance of COL–rGO hydrogels. (**b**) Cell viability of human dermal fibroblasts in the presence of rGO, collagen, COL hydrogel, COL/rGO_25_, COL/rGO_50_, and COL/rGO_100_. Cell migration assays in HDF. (**c**) Rate of wound closure and (**d**) Optical microscopy images of wound closure at 0, 24, 36, and 48 h of contact with the COL hydrogel, COL/rGO_25_, COL/rGO_50_, and COL/rGO_100_. The asterisk indicate a significant difference (* *p* < 0.05) when analyzed by Tuckey test by one-way ANOVA analysis.

The in vitro biodegradability of the hydrogels was evaluated using collagenase I, as shown in Figure 3a. During the first three hours, a substantial increase in the degradability rate was observed, being similar for all samples. This is due to the high concentration of collagenase I in the medium. Moreover, by increasing the rGO concentration, a higher resistance to biodegradation was observed, probably due to bigger interactions between collagen and rGO that strengthen the structure of the hydrogel matrix. Between 18 and 24 h of immersion, a decrease in the degradation rate was observed (1.1, 1.1, 0.9, and 0.2% for COL hydrogel, COL/rGO_25_, COL/rGO_50_, and COL/rGO_100_, respectively). It can be assumed that the interaction between rGO and the surrounding polymer chains created physical barriers that restricted the access of collagenase type I to the hydrogel network, thereby effectively reducing the degradation rate. The absence of biodegradation supports the conclusion that their stability in this medium is primarily due to their synthetic composition. This finding is crucial for the development of hydrogels for various biomedical applications where long-term stability and minimal degradation are desired.

In other studies where collagen-based hydrogels were synthesized, degradation rates of 50–60% were obtained using the same dose of cross-linking agent [65]. Maximum degradation obtained for COL–rGO hydrogels was 25%, so it can be concluded that the polymerization of dopamine and the enzymatic cross-linking used also provide a protective effect against biodegradation activity.

Wound dressings should possess different qualities, such as biocompatibility, non-toxicity, and the ability to facilitate cell migration [39]. The biocompatibility of all synthesized hydrogels exhibited a rate higher than 80% (Figure 3b). The viability of the pure collagen obtained was higher compared to that of the control (DMEM supplemented with 10% FBS and 1% antibiotic) due to its natural presence in human body tissues and excellent compatibility with the biological environment [66]. Moreover, it possesses a stable chemical structure that contains arginine–glycine–aspartic acid sites, which favor cell adhesion and proliferation [67,68]. rGO had a cell viability rate of 98.5 ± 4.3%. Previous research has indicated that the removal of oxygenated functional groups enhances cell biocompatibility, and the reduction achieved with DA does not pose a risk, in comparison with other reduction techniques that negatively affect cell viability [16,69].

The rGO incorporation into the matrix had no toxic impact and, in fact, promoted the survival of cells. Previous research has demonstrated that the water retention capacity of the hydrogel is heightened through the incorporation of rGO, leading to enhanced hydration and support for cellular interactions and viability [70]. Moreover, the roughness of the material has been found to facilitate interactions between functional groups and cells, potentially activating molecules responsible for cell adhesion and proliferation [71], an effect that was also observed for this study.

### 2.7. In Vitro Wound Healing Assay (Scratch Test)

Fibroblasts play a crucial role in the tissue repair process due to their ability to migrate and proliferate [72]. The ability of cells to migrate was evaluated by measuring the closure of a wound created in a fully confluent monolayer of fibroblast (Figure 3c). The optical microscopy images (Figure 3d) indicate the migration of fibroblast cells towards the scraping zone. It can be observed that this migration occurred after 24 h of incubation with COL/rGO_50_ and COL/rGO_100_ samples, and after 48 h for COL and COL/rGO_25_ samples. Subsequently, an accelerated migration of cells was observed, which was attributed to the elevated density of negative charges detected on the hydrogel’s surface. Various studies have reported that biomaterials with negative charges promote the proliferation stage in the process of wound healing, facilitating the migration and proliferation of cells to effectively cover the injured area [73]. This observation is further supported by the wound closure rates determined in this study. In the first 24 h, no significant differences in wound closure rates were observed (Figure 3d). However, between 24 and 36 h, all tested hydrogels exhibited a substantial decrease in the torn area. In fact, the rGO inclusion in the matrix had a positive impact on the cell migration process. Specifically, the COL/rGO_100_ material showed a closure rate of 84.5% after 48 h of contact. The observed phenomenon can be associated with the increase in the hydrogel’s conductivity, enabling the regeneration of the skin’s intrinsic electrical fields. These fields stimulate signals that direct the movement of epithelial cells towards the wound site, facilitating an ordered cell migration process that promotes efficient tissue healing [74,75,76].

## 3. Conclusions

Novel biocomposite hydrogels, referred to as COL–rGO, were successfully developed using a combination of DA polymerization and enzymatic cross-linking. The presence of rGO had beneficial effects, increasing the hydrogel’s swelling capacity and conductivity. The enzymatic cross-linking process, using HPR/H_2_O_2_, did not exhibit any cytotoxic effects at the concentration tested. Moreover, all the samples demonstrated biocompatibility with the HDF. The increased conductivity of the composite material facilitated cell migration, resulting in a shorter wound closure time during the cell proliferation stages. The combined effect of enzymatic cross-linking and dopamine polymerization, along with the incorporation of rGO, provides hydrogels with remarkable resistance to collagenase type I enzymatic action. This unique approach offers a promising strategy for developing more stable and durable hydrogels, with modulable biodegradation rates, for biomedical applications, such as tissue engineering and drug delivery systems. Based on these findings, it can be concluded that the synthesized biocomposite hydrogels are effective as wound dressings. To validate these findings, further in vivo tests will be conducted in order to confirm the outcomes observed in vitro.

## 4. Materials and Methods

### 4.1. Materials

Graphite powder (Flake, 325 mesh) was purchased from Asbury Online (Asbury Carbons, Asbury, NJ, USA). Sulfuric acid (H_2_SO_4_, 98%), hydrochloric acid (HCl, 37% *v/v*), nitric acid (HNO_3_, 65%), potassium permanganate powder (KMnO_4_, 99.9%), hydrogen peroxide (H_2_O_2_, 30%)), phosphate buffered saline (PBS), sodium hydroxide (NaOH, 98%), tris(hydroxymethyl)aminomethane ((HOCH_2_)_3_CNH_2_,99.8%), and dopamine hydrochloride (DA-HCl, minimum 98%). Peroxidase from Horseradish (HRP, Type VI-A, ≥250 units/mg solid (using pyrogallol), 950–2000 units/mg solid (using ABTS)), and type I collagen (bovine Achilles tendon) were purchased from Sigma-Aldrich Company, (St. Louis, MO, USA). All reagents and solvents were analytical grade and used without further purification. Milli-Q^®^ (Millipore Sigma, Burlington, USA) water was used throughout the study.

### 4.2. Synthesis of Graphene Oxide (GO) and Reduced Graphene Oxide (rGO)

GO synthesis was carried out by the oxidation of natural graphite powder using the modified Hummers method [77]. In a typical example, 2.25 g of graphite were added to a mixture of 30 mL of H_3_PO_4_ and 270 mL of H_2_SO_4_. Once dissolved, 13.5 g of KMnO_4_ were slowly added and heated for 1 h at 40 °C. The mixture was then cooled to 25 °C, and the oxidation was stopped by adding H_2_O_2_ (60% *v/v*) until a color change from black to dark green was observed, without the presence of foam. Subsequently, it was centrifuged at 5000 rpm for 20 min and the precipitate was washed with HCl (10% *v/v*) until a light brown color was achieved. After that, three Milli-Q water washes were carried out, and it was centrifuged at 9000 rpm for 20 min. In this stage, the absence of chlorides was verified using AgNO_3_. Then, a wash with ethanol and several washes with Milli-Q water were carried out to eliminate traces of alcohol. The solution was then dialyzed for 72 h, in 12 kDa dialysis bags and in 5 L of Milli-Q water, with water changes every 24 h. The dialyzed solution was placed in a cryogenic bath for 2 h at −40 °C and lyophilized for 72 h [15].

GO reduction was performed by the method proposed by Xu et al. [17]. A solution of 0.24 g of tris(hydroxymethyl)aminomethane in 200 mL of Milli-Q water was prepared, to which 0.05 g of DA and 0.1 g of GO were added. Once the GO dispersion was achieved, the pH was adjusted to 8.5 with HCl (5%) and sonicated for 20 min in an ice bath. Then, it was heated at 60 °C for 24 h with constant and gentle stirring to induce the reduction process. Subsequently, the sample was filtered through Whatman^®^ No.1 filter paper and the retained solid was dialyzed in Milli-Q water for 72 h, with water changes every 24 h. When the dialysis was finished, the sample was filtered and dried at room temperature.

### 4.3. Hydrogels synthesis

The synthesis method for COL–rGO hydrogels was based on collagen self-assembly and DA polymerization. The synthesis started by dissolving 1 g of collagen type I in 100 mL of HCl (0.05 M). The obtained solution was stored at 4 °C until further use. The second stage of synthesis consisted of diluting 5 mL of this solution with 4 mL of PBS (pH = 5). Once the mixture was homogeneous, 10 mg of DA were added and plasticized with 5% glycerol. Then, rGO was added in different amounts (0, 25, 50, and 100 mg) under vigorous stirring until complete dispersion was achieved. Then, 0.5 mL of HRP (0.5 mg/mL) and 0.5 mL of H_2_O_2_ (0.1 M) were added to increase the reactivity of the hydroxyl groups associated with the benzene structure of DA and thus to increase the cross-linking density of the hydrogel [25,78]. The samples were poured into non-stick paper molds (4 × 4 × 1.5 cm) and incubated at 50 rpm and 40 °C for 15 h. Hydrogel sample in the absence of rGO is defined as COL hydrogel, whereas the three biocomposite hydrogel samples are named COL/rGO_25_, COL/rGO_50_, and COL/rGO_100_.

### 4.4. Biocomposite Hydrogel’s Characterization

The morphology and physicochemical aspects of GO, rGO, and COL–rGO composites were studied through scanning electron microscopy (SEM), Fourier transform infrared spectroscopy (FTIR), X-ray diffraction (XRD), X-ray photoelectron spectroscopy (XPS), thermogravimetric analysis (TGA), contact angle, and by examining the surface charge for dynamic light scattering (DLS). Swelling capacity and penetration mechanism were determined by immersing the material in PBS at different pH values. The conductivity of the samples was determined using the four-point method. A detailed characterization of the materials is included in the Supplementary Materials.

### 4.5. In Vitro Biodegradation of COL–rGO Hydrogel

The in vitro biodegradation of the COL–rGO hydrogel was evaluated using collagenase I in a phosphate-buffered saline (PBS) solution. The hydrogel samples were placed in the PBS solution containing collagenase I (1 U/mL) agitated at 100 rpm/min and temperature of 37 °C. At specific time intervals (1, 3, 6, 12, 24, and 72 h), the solution was centrifuged to separate any unreacted enzyme, and the resulting precipitates were lyophilized and weighted. A control group without the enzyme was also studied. The hydrogel degradation was quantified using the biodegradation rate (BR), which was calculated using Equation (1):

(1) $$BR(\%)=\frac{W_{0}-W_{l}}{W_{0}}\times 100,$$

where W_0_ is the original dry hydrogel weight and W_l_ is the weight of samples after freeze-drying at predetermined time.

### 4.6. Cytotoxicity Assay

Cytotoxicity was assessed by conducting the 3-(4,5-dimethylthiazol-2-yl)-2,5-diphenyltetrazolium bromide (MTT) assay on human dermal fibroblast (HDF) cell lines, which were obtained from adult human epithelial tissue (Sigma, Santiago, Chile). The hydrogels were cut into circular discs (28 mg, 3.4 ± 0.6 mm diameter) and together with the raw materials (collagen and rGO) were subjected to UV radiation for 30 min. After sterilization, samples were added to a 6-well plate containing 1 mL of DMEM (Dulbecco’s Modification Eagle’s Medium) for every 10 mg of material added. The samples were incubated for 24 h at 37 °C and the liquid extract was filtered using a 0.22 μm cellulose acetate filter (STARLAB, United Kingdom). Then, it was mixed with 5% (*v/v*) fetal bovine serum (FBS) and 1% (*v/v*) antibiotics (100 u/mL of penicillin and 100 u/mL of streptomycin). The cell density of the HDFs was adjusted to 10^4^ cells per well, and they were cultured in DMEM nutrient medium in 96-well plates at 37 °C with a 5% CO_2_ atmosphere until a monolayer was formed. Subsequently, the nutrient medium was removed from the wells, and 100 μL of the material extracts were added to each well. After 48 h of incubation at 37 °C in a 5% CO_2_ atmosphere, 20 μL of MTT (5 mg/mL) were added to each well and incubated for an additional 4 h. To dissolve the formazan crystals, 100 μL/well of DMSO were added. The absorbance was determined using a microplate reader (Biotek synergy 2, Agilent Technologies, Santa Clara, CA, USA) at a wavelength of 540 nm. The relative cell viability was calculated from Equation (2):

(2) $$Relative\;cell\;viability\;(\%)=\frac{A_{540}\;of\;treated\;cells}{A_{540}\;of\;control\;cells}\times 100,$$

where A_540_ is the absorbance measured at a wavelength of 540 nm.

### 4.7. In Vitro Wound Healing Assay (Scratch Test)

The healing assays were carried out using HDF cells, with 5·10^4^ cells/well being seeded in 24-well plates. The plates were supplemented with DMEM medium containing 10% SFB and 1% antibiotic. The cells were cultured at 37 °C in a CO_2_ atmosphere of 5% until they reached 100% confluence. Then, the HDF monolayer was rinsed with PBS and a scratch in the center was created manually using a sterile plastic tip. Following the mapping of the wounds, they were thoroughly washed with PBS to ensure the removal of any detached cells. Subsequently, each material sample was fixed within the CellCrown 24 inserts (Corning Incorporated, Pittston, PA, USA) and carefully placed in the designated wells of a 24-well plate without any contact with the surface. The progress of wound closure was observed using a light microscope (MOTIC AE31, Richmond, BC, Canada) up to 48 h. Finally, the images were analyzed using ImageJ 1.53e^®^ software (National Institutes of Health, Bethesda, MD, USA). The wound closure rates were calculated according to Equation (3):

(3) $$Rate\;of\;wound\;closure\;(\%)=\frac{A_{0}-A_{t}}{A_{0}}\times 100$$

where A_0_ is the initial wound area and A_t_ is the wound area after each time interval.

### 4.8. Statistical Analysis

The spectra obtained in the chemical characterization of the hydrogels were processed using OriginPro 10.0.5.157 ^®^ software (OriginLab Corporation, Northampton, MA, USA). Statistical analyses were conducted in Statgraphics Centurion XVII^®^ software (Statgraphics Technologies, Inc., The Plains, VA, USA) using multivariate analysis of variance (ANOVA) with a significance level of *p*-value ≤ 0.05. The means were compared using the Tukey test with a 95% confidence interval, and the results were reported as mean ± standard deviation.

## Figures and Tables

**Table 1:** Thermal analysis data for the hydrogel samples.

Sample	T_h_ ^1^ (°C)	T_i_ ^2^ (°C)	T_max_ ^3^ (°C)	T_b_ ^4^ (°C)	M_600_^5^ (%)
COL hydrogel	83	225	286	337	7.6
COL/rGO_25_	92	211	280	335	13.6
COL/rGO_50_	100	193	275	331	18.7
COL/rGO_100_	115	184	270	317	24.2

^1^ T_h_: the extrapolated onset temperature of the water loss peak, ^2^ T_i_: the extrapolated onset temperature of the thermal decomposition peak, ^3^ T_max_: the temperature at the maximum weight loss rate, ^4^ T_b_: the extrapolated final temperature of the DTG curve. ^5^ M_600_: the residual carbon content at 600 °C.

**Table 2:** Contact angle, conductivity, and surface charge.

Sample	Contact Angle (°)	Conductivity (mS/m)	Surface Charge (mV)
GO	61.8 ± 1.4	121.4 ± 12.3	−32.8 ± 2.3
rGO	84.5 ± 2.0	232.6 ± 10.6	−70.2 ± 5.4
COL hydrogel	50.6 ± 1.3 ^a^	20.27 ± 0.25 ^a^	4.5 ± 0.8 ^a^
COL/rGO_25_	54.1 ± 1.6 ^a^	24.61 ± 0.03 ^b^	−46.8 ± 1.1 ^b^
COL/rGO_50_	73.6 ± 2.6 ^b^	29.72 ± 0.61 ^c^	−65.2 ± 2.2 ^c^
COL/rGO_100_	76.4 ± 2.8 ^b^	39.57 ± 0.19 ^d^	−79.0 ± 3.1 ^d^

The letters a, b, c and d indicate significant statistical differences between samples for *p*-value < 0.05.

## Data Availability

All data and materials are available upon request from the corresponding author. The data are not publicly available due to ongoing research using a part of the data.

## Supplementary Materials

The following supporting information can be downloaded at: https://www.mdpi.com/article/10.3390/gels10070448/s1, Figure S1: Thermogravimetric analysis curve and Derivative thermogravimetric analysis of COL, COL/rGO_25_, COL/rGO_50_ and COL/rGO_100_; Table S1: The impact of medium pH on the surface charge of collagen.
