# Supplementary material for: Innovative Approach to Accelerate Wound Healing: Synthesis and Validation of Enzymatically Cross-Linked COL–rGO Biocomposite Hydrogels
Source: Gels. 2024 Jul 6;10(7):448. doi: 10.3390/gels10070448 (PMC11275597; doi:10.3390/gels10070448)
Supplement: Supplementary file 1 [file gels-10-00448-s001.zip › gels-3086945-supplementary.pdf]

gels

Supporting Information

Article

Innovative Approach to Accelerate Wound Healing: Synthesis and Validation of Enzymatically Cross-linked COL-rGO-PDA Biocomposite Hydrogels

Luisbel González [1], Víctor Espinoza [1], Mauricio Tapia [1], Valentina Aedo [1], Isleidy Ruiz [1], Manuel Meléndrez [2], Claudio Aguayo [3], Leonard I. Atanase [4,5,*] and Katherina Fernández [1,*]

1 Laboratorio de Biomateriales, Departamento de Ingeniería Química, Facultad de Ingeniería, Universidad de Concepción, Concepción, Chile; luisbgonzalez@udec.cl (L.G.); vespinozar@udec.cl (V.E.); mtapia2017@udec.cl (M.T.); vaedo2017@udec.cl (V.A.); isruiz@udec.cl (I.R.)
2 Departamento de Ingeniería de Materiales, Facultad de Ingeniería, Universidad de Concepción, Concepción, Chile; mmelendrez@udec.cl (M.M.)
3 Departamento de Bioquímica Clínica e Inmunología, Facultad de Farmacia, Universidad de Concepción, Concepción, Chile; caguayo@udec.cl (C.A.)
4 Faculty of Medicine, "Apollonia" University of Iasi, 700511 Iasi, Romania
5 Academy of Romanian Scientists, 050045 Bucharest, Romania
* Correspondence: leonard.atanase@yahoo.com; kfernandeze@udec.cl

1. Materials and methods

1.1 Material characterization

Scanning electron microscopy (SEM). The SEM analyzes were employed to examine the morphology of the GO, rGO, and collagen hydrogels. The hydrogels were subjected to freezing at -40 °C for 24 hours, followed by lyophilization at -80 °C for 48 hours. SEM images were obtained using a JEOL JSM-6380LV microscope from Japan, operating at 10 kV. The hydrogels were coated using a gold sputter coater, and their surfaces were subsequently observed at various resolutions.

Fourier Transform Infrared Spectroscopy (FTIR). The FTIR was used to investigate the chemical structure of the individual compounds and their interactions in the hydrogel. The spectra were recorded in the Perkin Elmer UATR Two FTIR Spectrometer. The wavenumber range analyzed was 4000-500 cm-1 and a total of 40 accumulated scans were realized.

X-ray diffraction (XRD). The X-ray diffraction (XRD) was used to determine the reduction degree of GO and the crystallinity of hydrogels. The X-ray measurements were conducted using a Bruke Axs D4 Endeavor diffractometer from the USA. The reference target used was Cu Kα radiation with a wavelength of 1.541841 Å and a power output of 2.2 kW. The voltage used was 40 kV, and the current was set at 20mA. The samples were measured within a range of 2 to 50° for 141 seconds, with increments of 0.02°.

X-ray photoelectron spectroscopy (XPS). XPS technique was employed to accurately determine the surface chemistry of the collagen, GO, rGO compounds, and the COL and COL/rGO_{50} hydrogels. The measurements were conducted using a Surface Analysis Station 1 (STAIB model RQ300/2, USA) under ultra-high vacuum conditions (< 10-9 bar). The station was equipped with a hemispherical electron analyzer (SPEC PHOIBOS 100, Germany). The photoelectrons were stimulated by non-monochromatic radiation from

Mg Kα (1486.6 eV) and analyzed with a fixed energy step of 1 eV. A 300 W X-ray source was employed.

Thermogravimetric analysis (TGA). TGA technique was used to evaluate the thermal stability of hydrogels. The measurements were performed using a Cahn-Versatherm thermogravimetric analyzer, with a sensitivity of 0.1 μg. The hydrogels were subjected to a heating rate of 10°C/min, under a nitrogen atmosphere (flow rate: 100 mL/min), within a temperature range of 30°C to 800°C.

Contact angles of the sample were determined using a Goniometer Drop Shape Analyzer DSA-25 (Krüss, Germany). Deionized water was dispensed onto the sample using a microsyringe needle, and a picture of the resulting droplet was taken. The software examined the droplet's shape by capturing photos at 30 ms intervals. To reduce errors from material absorption, the initial measurement value was used. The baseline was manually adjusted to mitigate the impact of material roughness. Each material underwent 5 repetitions for accuracy.

Contact angle measurement for GO, rGO and pure collagen was determined using the Washburn method [28]. Capillary rise is based on Poiseuille's law for a flow of liquid rising through a porous medium (equation S.1).

$$\nu = \frac{{R_D}^2}{8\eta} \cdot \frac{\Delta P}{l} \qquad (S.1)$$

Where v is the kinetics of flow, RD the mean hydrodynamic radius of the capillary, η the viscosity of the liquid, l the length of the capillary and ΔP the pressure difference. Considering that ΔP is the capillary pressure and neglecting the hydrostatic pressure, it can be integrated with boundary conditions for zero height and time, where the Washburn equation (equation S.2) is obtained.

$$h^2 = \frac{r\gamma l\cos\theta}{2\eta} t \qquad (S.2)$$

Where r is hair cell radius, γ liquid surface tension, θ contact angle and t time. Considering that the porous medium is a bunch of capillaries of constant radius, the height-related mass can be monitored ($w = \varepsilon\rho\pi R^2 h$), where ε is the porosity of the packed powder column, ϱ the density of the liquid and R the inner radius of the tube. By combining the above equations, an equation can be obtained that describes the mass of liquid ascended as a function of time. The packing properties are considered in the conformational constant of the porous medium c, $w = r\varepsilon^2(\pi R^2)^2$.

$$w^2 = c\frac{\rho^2\gamma l cos\theta}{2\eta} t \qquad (S.3)$$

The packaging was made up of 2g of pulverized and homogenized sample. The determination of the structural constant of the packing was carried out in triplicate using hexane and considering perfect wettability. The w^2 vs t curves were obtained on a SIGMA KSV 700 tensiometer (Sigma Elektro GmbH, Germany) using the "Attension Sigma" software and the "Powder" test.

The surface charge was determined through ζ-potential measurements using the Dynamic Light Scattering principle (SZ-100 Nano particle analyzer, Horiba Scientific, Japan). The hydrogel samples (2.0 cm^2) were dissolved in Milli-Q® water at pH 6.5, and then shaken and sonicated for 20 minutes to ensure uniformity. The measurements were performed in quintuplicate to ensure accuracy and reproducibility.

Swelling capacity and penetration mechanism

Firstly, the hydrogels were submerged in PBS buffer solutions with varying pH levels (2, 4, and 6). The temperature was maintained at a constant 25 ºC throughout the experiment. The immersion time was varied between 1, 2, 3, 4, 5, 10, 15, 20, 30, and 50 min. After each immersion, any excess water was eliminated by using filter paper, and

the weight of the hydrogels was promptly recorded. The swell ratio (SR) was calculated using Equation S.4:

$$SR\ (\%) = \frac{W_{wet} - W_{dry}}{W_{dry}} \times 100 \qquad (S.4)$$

where W_{dry} is the dry sample weight, and W_{wet} is the wet sample weight (after contact with the liquid medium).

To determine the nature of the diffusion inside the hydrogel, the semi-empirical equation developed by Peppas was used [29]:

$$\alpha = \frac{m_t}{m_\infty} = kt^n \qquad (S.5)$$

where m_t and m_∞ are the absorbed mass at time t and at equilibrium respectively, k is the characteristic constant related to the lattice structure of the gels and n is the diffusion exponent. The description of the penetration mechanism was made for $m_t/m_\infty \leq 0.6$ [30].

The conductivity of the samples was determined using the four-point method. A Keithley 2450 Source Meter Instrument was employed, connected to two adjustable Teflon plates fitted with 4 AC175 clamps (Fluke, USA). The plates were connected to the instrument using 4 TL78 leads (Fluke, USA). The central part of the plate had a pogo pin, measuring 10.5 mm in length and 7 mm in height, which consisted of 4 electrodes spaced 2.54 mm apart. For the measurement, square hydrogels measuring 2 x 2 cm with uniform thickness were fabricated. The conductivity (σ) was calculated using Equation S.6:

$$\sigma = \frac{1}{\rho} = \frac{L}{R \cdot S} \qquad (S.6)$$

where ϱ is the resistivity of the sample, R is the resistance of the sample, and S and L represent the cross-sectional area and length of the sample, respectively [31].

Table S1: The impact of medium pH on the surface charge of collagen

pH	Surface Charge (mV)
4	12.3 ± 0.3
5	9.2 ± 0.4
6	5.4 ± 0.2
7	1.4 ± 0.2
8	-8.3 ± 0.8
9	-13.5 ± 0.2

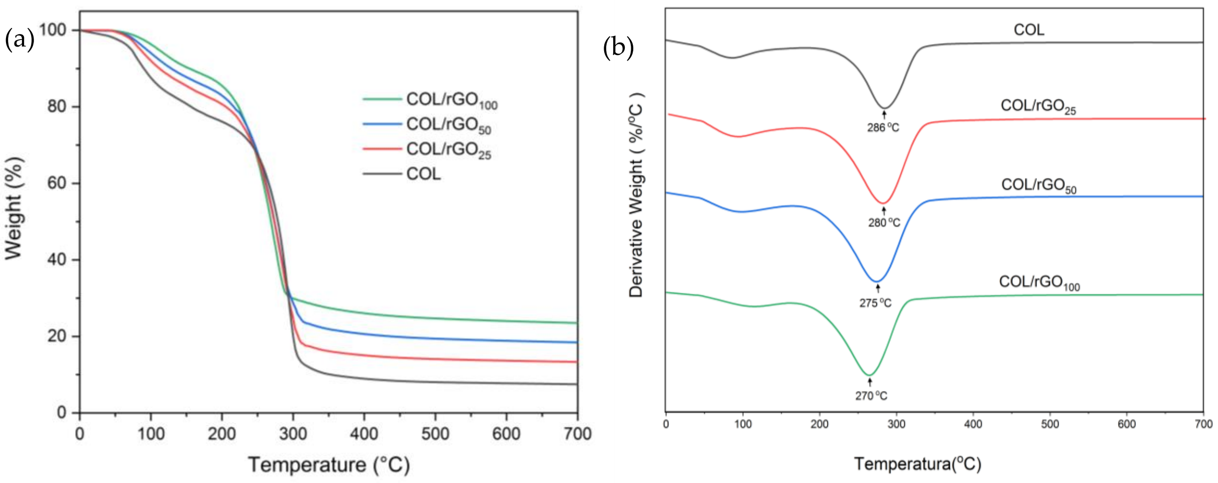

Figure S1. (a) Thermogravimetric analysis curve and (b) Derivative thermogravimetric analysis of COL, COL/rGO25, COL/rGO50 and COL/rGO100.
